# 20-Hydroxyecdysone-Responsive *miR*-*2788* Regulates the Larval–Pupal Transition by Targeting Trehalase in *Galeruca daurica*

**DOI:** 10.3390/insects17050502

**Published:** 2026-05-14

**Authors:** Mingze Shan, Haichao Wang, Yan Zhao, Baoping Pang, Ling Li, Yanyan Li, Haibin Han

**Affiliations:** Research Center for Grassland Entomology, Inner Mongolia Agricultural University, Hohhot 010020, China; 15304757811@163.com (M.S.); wanghc@imau.edu.cn (H.W.); lyy4455@163.com (Y.Z.); pangbp@imau.edu.cn (B.P.); lling@imau.edu.cn (L.L.)

**Keywords:** *Galeruca daurica*, 20-hydroxyecdysone, *miR*-*2788*, *Treh1*, chitin biosynthesis, metamorphosis, trehalose metabolism

## Abstract

Metamorphosis in insects is regulated by hormonal signals and metabolic pathways. In this study, we characterized a 20E-mediated *miR*-*2788*/*Treh1* regulatory axis involved in the larval-to-pupal transition in the leaf beetle *Galeruca daurica*. Dual-luciferase assays and expression profiling suggest that *miR*-*2788* directly targets and suppresses trehalase 1 (*Treh1*), a key enzyme for trehalose hydrolysis. We found that exogenous 20E treatment significantly downregulated *miR*-*2788*, leading to the derepression of *Treh1* and increased expression of chitin biosynthetic genes (*CHI*, *CHS*, and *GPI*). Conversely, *miR*-*2788* overexpression or *Treh1* silencing via RNAi resulted in massive trehalose accumulation, reduced chitin synthesis, and lethal developmental arrest during the metamorphic transition. Furthermore, pharmacological inhibition of *Treh1* with Validamycin not only recapitulated these chitin-deficiency phenotypes but was also associated with an upregulation of *miR*-*2788*. These results describe a signaling cascade where 20E modulates the conversion of carbohydrate to chitin through the *miR*-*2788*/*Treh1* axis. This study advances our understanding of hormonal–miRNA–metabolic crosstalk and identifies *miR*-*2788* as a potential target for RNAi-based biopesticide development.

## Simple Summary

The leaf beetle *Galeruca daurica* is a destructive pest of desert grasslands, causing significant ecological and economic damage. Its survival depends on a process called metamorphosis—the transformation from larva to pupa—which is associated with the precise production of chitin. In this study, we identified a molecular axis involving a small RNA molecule (*miR*-*2788*) and a key metabolic enzyme (*Treh1*). We found that the molting hormone 20E contributes to the suppression of *miR*-*2788*, thereby promoting the activation of *Treh1* to turn stored sugar (trehalose) into chitin. When this pathway is disrupted by chemical inhibitors, the beetles develop severe deformities and die. These findings provide theoretical insights for developing eco-friendly molecular strategies to manage this pest by specifically sabotaging its growth.

## 1. Introduction

Insect metamorphosis is a complex physiological remodeling process characterized by the histolysis of larval tissues and the subsequent reconstruction of pupal or adult organs [1,2]. For holometabolous insects, the larval–pupal transition is a critical developmental checkpoint that determines survival and evolutionary success, a process regulated by the endocrine system and various metabolic pathways [3,4,5,6]. Among these, chitin, the core structural component of the insect exoskeleton, undergoes periodic synthesis, assembly, and degradation, which serves not only as the material foundation for successful molting but also as a vital barrier ensuring mechanical strength and stress resistance [7,8,9]. Previous studies have demonstrated that disrupting the chitin biosynthesis pathway frequently leads to molting obstacles, developmental deformities, or lethality. Consequently, this pathway is considered a potential target for the development of intervention strategies [10,11,12].

The biosynthesis of chitin is an energetically intensive physiological activity with substantial precursor demands, and its carbon metabolism is closely associated with carbohydrate catabolism [13]. In the insect circulatory system, trehalose is the primary hemolymph sugar and serves as a major glucose donor for chitin synthesis [14]. Trehalase (Treh), the sole and irreversible rate-limiting enzyme that catalyzes the hydrolysis of trehalose into two glucose molecules, is involved in providing glucose for the chitin biosynthetic pathway, such as the hexosamine biosynthetic pathway [15,16]. It has been reported in multiple insect species that during the peak of pupation, trehalase expression levels are associated with chitin deposition rates [17,18]. However, while the classic enzymatic steps of chitin synthesis are well-characterized, the underlying molecular mechanisms by which the organism coordinates trehalose metabolism via post-transcriptional mechanisms to meet the instantaneous chitin demand during the volatile window of metamorphosis remain to be further explored in specific insect groups.

20-hydroxyecdysone (20E) is a key hormonal signal driving insect metamorphosis, initiating the process through a cascade of transcription factor inductions [19,20]. Recently, microRNAs (miRNAs) have emerged as important post-transcriptional regulators, acting as molecular buffers between complex hormonal signals and specific metabolic outputs [21,22,23]. For instance, conserved *miR*-*8-3p* and *miR-2a-3* have been shown in Hemipteran pests to regulate chitin homeostasis during molting by targeting trehalase and phosphoacetylglucosamine mutase (PAGM) [24]. While miRNA-mediated regulation of trehalose metabolism has been investigated in Lepidoptera [25], the specific role and hormonal regulation of these axes in Coleoptera remain less understood [26,27].

The leaf beetle *Galeruca daurica* Joannis is an outbreak pest of the Inner Mongolian grasslands, characterized by aggregated larval feeding and highly synchronized pupation, making it exceptionally sensitive to chitin synthesis inhibitors [28,29]. This study aims to investigate the potential role of 20E-responsive *miR*-*2788* in regulating the larval–pupal transition of *G. daurica*. We verified the direct targeting of the soluble trehalase gene (*Treh1*) by miR-2788 via dual-luciferase reporter assays. We further discovered that the 20E peak is associated with the suppression of *miR*-*2788* expression, thereby contributing to the derepression of *Treh1* to support the glucose supply required for pupation. This research contributes to our understanding of hormone-mediated metabolic reprogramming but also provides a theoretical foundation for potential pest management strategies.

## 2. Materials and Methods

### 2.1. Insect Rearing

Eggs and pupae of *G. daurica* were collected from the grasslands of Siziwang Banner (41° N, 112° E), Inner Mongolia, China, from August to September 2024. For developmental expression profiling, the eggs were reared indoors, and the resulting first-instar, second-instar, and third-instar larvae, along with the field-collected pupae, were used as samples. Since it is difficult for this species to complete its lifecycle under laboratory conditions, the third-instar larvae used for microinjection experiments were obtained from second-instar larvae collected from the same field site in May 2024, which were then reared indoors to the third instar. All insects were maintained in a climate-controlled chamber at 25 ± 1 °C with a relative humidity of 70% ± 5% and a photoperiod of 14:10 h (L:D). Larvae were reared in plastic containers and provided with fresh Allium tuberosum Regel.

### 2.2. Targeted Binding Site Prediction and Luciferase Reporter Construction

The physical interaction between *miR*-*2788* and its putative target, *Treh1*, was computationally predicted using the miRanda and RNAhybrid algorithms, with an energy threshold set at MFE ≤ −20 kcal/mol. For experimental validation, a cDNA segment of the *Treh1* coding sequence containing the predicted *miR*-*2788* recognition element was synthesized. This fragment was terminally tagged with Sac I and Xho I linkers and directionally subcloned into the pmirGLO Dual-Luciferase vector (Promega, WI, USA) to generate the wild-type reporter (*Treh1*-WT). A corresponding mutant reporter (*Treh1*-MUT) was engineered by replacing the complementary seed-match nucleotides within the binding site.

HEK293T cells were maintained in DMEM medium (Sigma, Shanghai, China) supplemented with 10% FBS (KEL, Shanghai, China). For the assay, cells were harvested and seeded into 12-well culture plates (Corning, NY, USA). Once the cell density reached approximately 80% confluence, co-transfection was initiated. Each well received a mixture containing 1 μg of reporter plasmid and 100 pmol of *miR*-*2788* mimics (or NC mimics), facilitated by GP-transfect-Mate reagent (GenePharma, Suzhou, China). The medium was replaced with fresh DMEM 6 h post transfection. After a 24 h incubation period, luciferase activities were quantified using the Dual-Luciferase Reporter Assay System (Promega) on a Synergy HTX multi-mode reader. Firefly luciferase levels were normalized against Renilla luciferase activity to determine the relative reporter expression.

### 2.3. Hormonal Induction and Downstream Metabolic Assays

To investigate the endocrine regulation of the *miR*-*2788*-*Treh1* axis during metamorphosis, early 3rd-instar larvae were microinjected with 1 μL of 20-hydroxyecdysone (20E) at a concentration of 2.5 μg/μL, while the control group received an equivalent volume of DMSO diluted in saline. Integrated samples were harvested at 6, 12, and 24 h post injection to determine the transcriptional response of the regulatory axis via qRT-PCR. Subsequently, at 48 h post treatment, the potential consequences on carbohydrate metabolism and chitin deposition were evaluated. Trehalose and glucose contents were measured using specific enzymatic detection kits (Solarbio, Beijing, China) following the manufacturer’s protocols. Total chitin was extracted from the larval cuticle and quantified according to established methods. To further assess the metabolic response, the transcript levels of downstream rate-limiting genes, including *CHS*, *CHI*, and *PAGM*, were systematically quantified.

### 2.4. In Vivo Functional Analysis of the miR-2788-Treh1 Axis

To evaluate the physiological significance of the identified regulatory axis during metamorphosis, functional interventions including miRNA manipulation and RNA interference (RNAi) were performed on late 3rd-instar larvae. Specifically, individuals were microinjected with 1 μL of 40 μM *miR*-*2788* agomir or antagomir to achieve transient overexpression or knockdown of the miRNA, respectively. For the depletion of the target gene, double-stranded RNA (dsRNA) targeting *Treh1* (*dsTreh1*) was synthesized in vitro using the T7 RiboMAX™ Express RNAi System (Promega), and 1 μg of *dsTreh1* was delivered per larva, with *dsGFP* serving as the negative control. Following these treatments, the larvae were monitored daily to determine the pupation and adult emergence rates. Any phenotypic manifestations of developmental asynchrony, molting failure, or pupal deformities were meticulously recorded and photographed to assess the impact of the *miR*-*2788*-*Treh1* axis on larval–pupal transition.

### 2.5. Validamycin Treatment

To investigate the pharmacological impact of trehalase inhibition on the *miR*-*2788*/*Treh1* axis, Validamycin was administered via microinjection. Third-instar 3-day-old (3L3d) larvae of *G. daurica* with uniform body size were selected and injected with 0.8 μL of Validamycin solution using a microinjector. An equal volume of PBS solution was injected as the solvent control. In addition, 3-day-old adults (both males and females) were separately injected with 1 μL of Validamycin solution, with PBS injection serving as the control. Each treatment was performed with three biological replicates, each consisting of five individuals (pooled for RNA extraction). At 48 h post injection, samples were rapidly frozen in liquid nitrogen and stored at −80 °C.

### 2.6. RNA Extraction, qRT-PCR, and Statistical Analysis

For all bioassays, three independent biological replicates were performed, with at least 30 larvae per replicate to ensure statistical consistency. Total RNA was isolated from treated larval tissues using RNAiso Plus (Takara, Dalian, China), and genomic DNA contamination was eliminated prior to cDNA synthesis. For mRNA and miRNA quantification, reverse transcription was performed using the PrimeScript RT Reagent Kit and Mir-X miRNA First-Strand Synthesis Kit (Takara), respectively. Quantitative real-time PCR (qRT-PCR) was conducted to measure the relative abundance of *miR*-*2788* and its associated target genes, with the small nuclear RNA U6 and succinate dehydrogenase complex subunit A (SDHA) utilized as internal references for normalization [30]. Data were first tested for normality using the Shapiro–Wilk test and for homogeneity of variance using Levene’s test. For datasets that met the assumptions of normality and equal variance, statistical significance was determined using Student’s *t*-test for pairwise comparisons, or one-way analysis of variance (ANOVA) followed by Duncan’s post hoc test. For datasets that did not meet these assumptions (e.g., non-normal distribution or heterogeneity of variance), the non-parametric Mann–Whitney U test was used. All statistical analyses were performed using SPSS 20.0, and exact *p*-values are reported for all significant differences (*p* < 0.05). The primer sequences used in this study are listed in Supplementary Table S1.

## 3. Results

### 3.1. Target Validation and Developmental Expression Profiling of the miR-2788-Treh1 Axis

**Figure 1:**
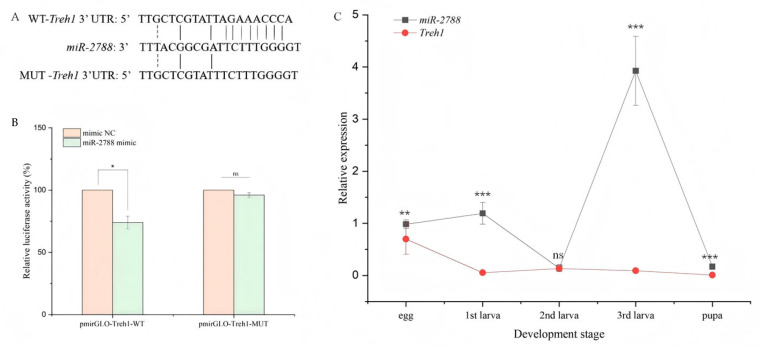
Target validation and spatiotemporal expression profiling of *miR*-*2788* and *Treh1* in *G. daurica*. (**A**) Schematic representation of the predicted binding site between *miR*-*2788* and *Treh1*. (**B**) Relative luciferase activity in HEK293T cells co-transfected with *miR*-*2788* mimics and reporter vectors (WT or MUT). (**C**) Developmental expression profiles of *miR*-*2788* and *Treh1* across different life stages. Relative expression levels of *miR*-*2788* and *Treh1* were normalized to U6 and SDHA, respectively, using quantitative real-time polymerase chain reaction (qRT-PCR). *, **, and *** represent a significant difference at *p* < 0.05, 0.01, and 0.001, while “ns” indicates no significant difference respectively, by Student’s *t* test. Bars represent the means ± SE. (The same applies below.)

To investigate the regulatory mechanism of *Treh1*, in silico prediction was initially performed, identifying a conserved binding site for *miR*-*2788* within the *Treh1* coding sequence (Figure 1A). The direct biochemical interaction was quantified using a dual-luciferase reporter system in HEK293T cells. As illustrated in Figure 1B, co-transfection of *miR*-*2788* mimics with the wild-type *Treh1* reporter resulted in a significant reduction in luciferase activity, which decreased to approximately 0.74-fold of that in the negative control (mimic NC) group (*p* < 0.05). Conversely, this inhibitory effect was completely abolished when the binding site was mutated, with the relative luciferase activity of the MUT group showing no significant difference compared to the control. These results indicate that *miR*-*2788* can negatively regulate *Treh1* expression by specifically binding to its CDS region. Both *miR*-*2788* and Treh1 have basal expression during the larval stage, but the expression level of *miR*-*2788* is higher than that of Treh1. The expression level of *miR*-*2788* shows an upward trend after the first larval instar, then decreases to the lowest level at the second larval instar, reaches the highest level at the third larval instar, and then rapidly decreases during the pupation stage. The expression level of *Treh1* is significantly lower than that of *miR*-*2788*, and the expression levels during the entire expression period are opposite to each other (Figure 1C). The results indicate that the *miR*-*2788* and *Treh1* genes exhibit a negative regulatory pattern during the developmental process from the egg stage to the pupation stage.

### 3.2. In Vivo Validation of miR-2788-Mediated Regulation of Treh1

To verify whether *miR*-*2788* modulates *Treh1* expression within the larval body of *G. daurica*, we performed gain- and loss-of-function experiments by microinjecting *miR*-*2788* agomir (Ago *miR*-*2788*) and antagomir (Ant *miR*-*2788*) into third-instar third-day (3L3d) larvae. Based on our laboratory’s optimized protocols, the regulatory effects were assessed at 48 h post injection.

**Figure 2:**
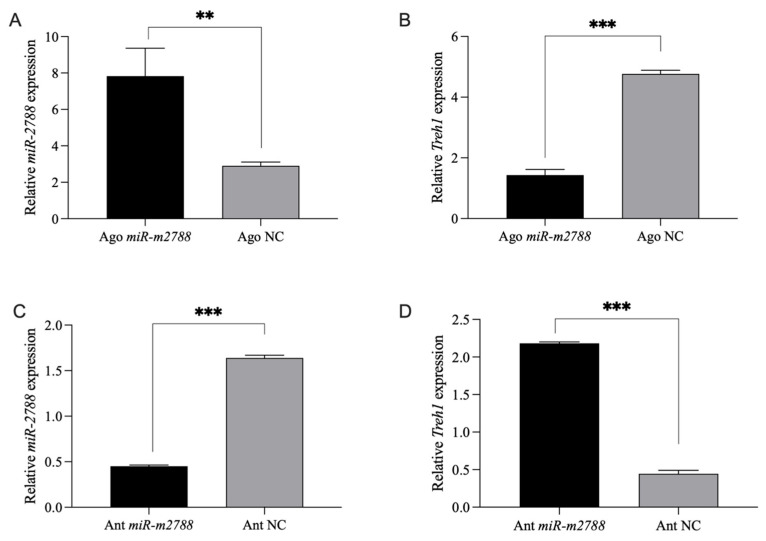
In vivo regulatory relationship between *miR*-*2788* and its target gene *Treh1*. (**A**) Relative expression levels of *miR*-*2788* in 3rd-instar 3rd-day (3L3d) larvae of *G. daurica* following Ago *miR*-*2788* treatment. Ago *miR*-*2788* (M = 7.83, SD = 4.35); Ago NC (M = 2.91, SD = 0.58), t(7.25) = 3.17, *p* = 0.0068. (**B**) Relative expression levels of *Treh1* in 3L3d larvae following Ago *miR*-*2788* treatment. Ago *miR*-*2788* (M = 1.43, SD = 0.37); Ago NC (M = 4.77, SD = 0.24), t(6) = −15.20, *p* < 0.001. (**C**) Relative expression levels of *miR*-*2788* in 3L3d larvae following Ant *miR*-*2788* treatment. Ant *miR*-*2788* (M = 0.45, SD = 0.03); Ant NC (M = 1.64, SD = 0.06), t(4.17) = −36.91, *p* < 0.001. (**D**) Relative expression levels of *Treh1* in 3L3d larvae following Ant *miR*-*2788* treatment. Ant *miR*-*2788* (M = 2.18, SD = 0.04); Ant NC (M = 0.44, SD = 0.09), t(6) = 34.57, *p* < 0.001. All data are presented as mean ± SE. Statistical significance was analyzed using Student’s *t*-test or Mann–Whitney U test; ** and *** represent the significant difference at *p* < 0.01 and *p* < 0.001. These conventions are applied throughout this study.

The qRT-PCR results demonstrated that the administration of Ago *miR*-*2788* led to a massive enrichment of *miR*-*2788* levels in the larvae, which was accompanied by a significant downregulation of *Treh1* mRNA transcripts (Figure 2A,B). This confirmed that exogenous elevation of *miR*-*2788* suppresses the expression of its target gene in vivo.

Conversely, the injection of Ant *miR*-*2788* resulted in a substantial reduction in endogenous *miR*-*2788* abundance. This depletion effectively derepressed the target gene, as evidenced by a significant and compensatory increase in *Treh1* expression levels (Figure 2C,D). Taken together, these reciprocal results from both agomir and antagomir treatments provide conclusive evidence that *miR*-*2788* acts as a potent negative regulator of *Treh1* during the critical larval-to-pupal transition of *G. daurica*.

### 3.3. Metabolic and Phenotypic Consequences of miR-2788 Manipulation

To further evaluate how *miR*-*2788* participates in metamorphosis via its target *Treh1*, we analyzed the expression of key enzymes in the chitin biosynthetic pathway, carbohydrate levels, and developmental phenotypes at 48 h post injection of *miR*-*2788* agomir and antagomir.

**Figure 3:**
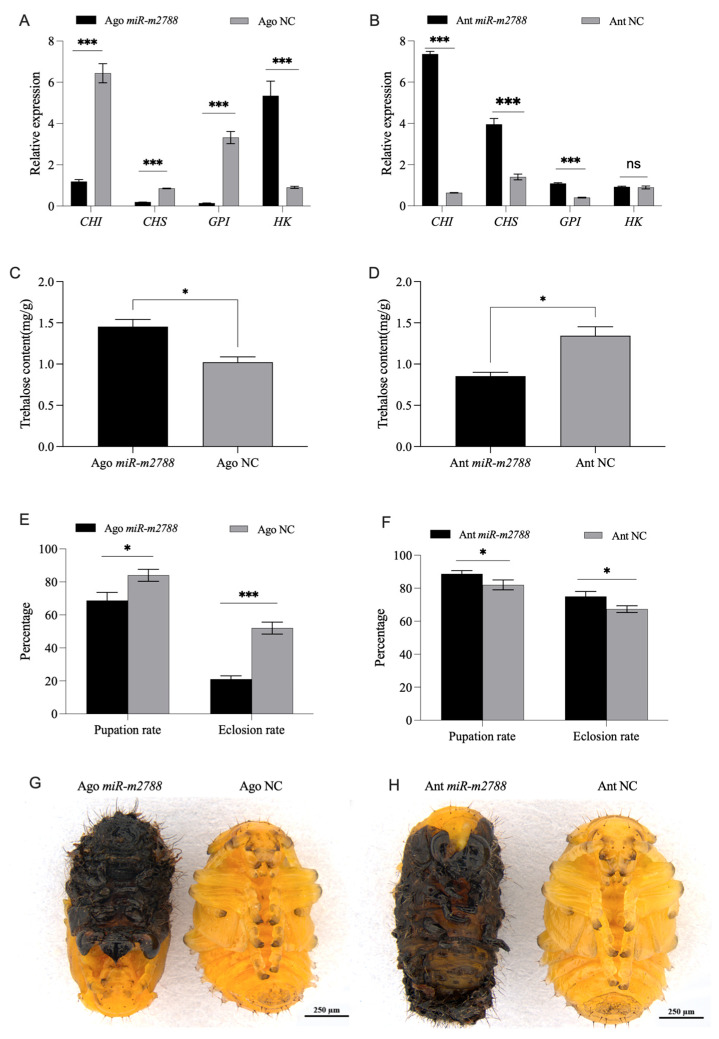
Effects of *miR*-*2788* on chitin biosynthesis, trehalose metabolism, and pupation success. (**A**,**B**) Relative expression levels of key chitin biosynthetic genes (*CHI*, *CHS*, *GPI*, and *HK*) following *miR*-*2788* agomir and antagomir treatment. (**C**,**D**) Changes in endogenous trehalose content following Ago *miR*-*2788* and Ant *miR*-*2788* injections, respectively. (**E**,**F**) Statistical analysis of pupation and adult emergence rates in different treatment groups. (**G**,**H**) Representative images of developmental deformities and molting failure induced by Ago *miR*-*2788* treatment. Relative expression levels of *miR*-*2788* and *Treh1* were normalized to *U6* and *SDHA*, respectively, using quantitative real-time polymerase chain reaction (qRT-PCR). * and *** represent the significant difference at *p* < 0.05 and 0.001, while “ns” indicates no significant difference, respectively, by Student’s *t* test. Bars represent the means ± SE.

The qRT-PCR analysis revealed that exogenous overexpression of *miR*-*2788* (Ago *miR*-*2788*) significantly suppressed the transcript levels of core chitin biosynthetic genes, including *CHI*, *CHS*, and *GPI* (Figure 3A). Conversely, hexokinase (*HK*) expression was markedly upregulated, potentially suggesting a metabolic compensatory response to the excessive accumulation of trehalose (Figure 3A). In the antagomir-treated group, the expression of *CHI*, *CHS*, and *GPI* was significantly elevated (Figure 3B).

Corresponding to these transcriptional changes, trehalose metabolism exhibited a strong responsive shift. *miR*-*2788* overexpression led to a significant increase in internal trehalose content (Figure 3C), whereas its silencing via antagomir resulted in a marked reduction in trehalose levels (Figure 3D), suggesting that *miR*-*2788* is involved in regulating trehalose hydrolysis.

At the physiological level, *miR*-*2788* manipulation caused severe developmental defects during the larval–pupal transition. Larvae treated with Ago *miR*-*2788* exhibited failed or incomplete pupation, and approximately 26% of them resulted in black and deformed pupae (Figure 3G). This melanization may stem from the impaired chitin biosynthesis and subsequent integumentary abnormalities caused by the reduced glucose supply after *Treh1* suppression, which potentially triggers a systemic stress or immune response. The pupation rate in the Ago *miR*-*2788* group dropped significantly to 68.8% (compared to 84% in the control), representing an 18.1% decrease. More critically, the adult emergence rate decreased to 21%, which was only 35.6% of the control rate (59% reduction) (Figure 3E).

In contrast, Ant *miR*-*2788* treatment appeared to facilitate development, as evidenced by a slight increase in both the pupation rate (88.6% vs. 82% in control) and the emergence rate (75% vs. 67% in control), although a small fraction of individuals still displayed minor pupation irregularities (Figure 3H,F). These dual-directional results demonstrate that the precise regulation of the *miR*-*2788*/*Treh1* axis is important for maintaining chitin homeostasis and ensuring successful metamorphosis in *G. daurica*.

### 3.4. Functional Validation of Treh1 via RNAi

To evaluate whether the developmental and metabolic disruptions observed in *miR*-*2788*-overexpressing larvae were associated with the suppression of its target, *Treh1*, we employed RNA interference (RNAi) to directly silence *Treh1* expression.

**Figure 4:**
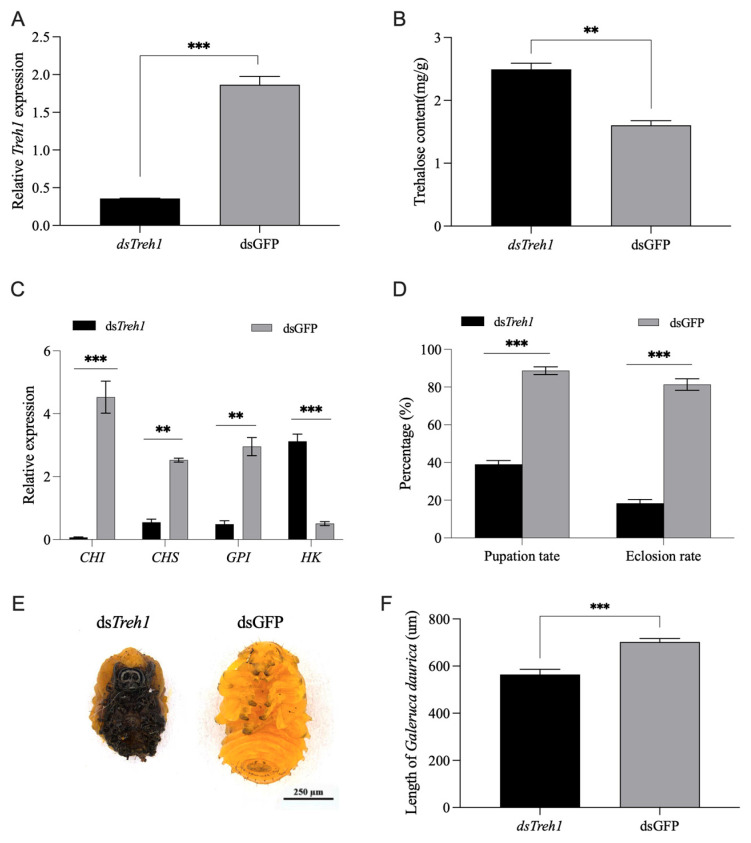
Silencing of *Treh1* by RNAi mimics the developmental defects induced by *miR*-*2788*. (**A**) Interference efficiency of *dsTreh1* on the relative expression of *Treh1* at 48 h post injection. *dsTreh1* (M = 0.36, SD = 0.01); *dsGFP* (M = 1.87, SD = 0.22), t(3.02) = −13.81, *p* < 0.001. (**B**) Changes in endogenous trehalose content following *Treh1* silencing. (**C**) Transcriptional response of downstream chitin biosynthetic pathway genes (*CHI*, *CHS*, and *GPI*) and the compensatory gene (*HK*) after RNAi. (**D**) Statistical analysis of pupation and adult emergence rates in dsTreh1-treated larvae compared to the *dsGFP* control. (**E**) Representative images showing impaired larval–pupal metamorphosis and reduced pupal size following *Treh1* knockdown. (**F**) Statistical analysis of the body lengths of the larvae treated with *dsTreh1* and the larvae in the *dsGFP* control group. *dsTreh1* (M = 564.22, SD = 33.45); *dsGFP* (M = 702.02, SD = 33.45) t(8) = −5.12, *p* < 0.001. Relative expression levels of *miR*-*2788* and *Treh1* were normalized to *U6* and *SDHA*, respectively, using quantitative real-time polymerase chain reaction (qRT-PCR). ** and *** represent the significant difference at *p* < 0.01 and 0.001, by Student’s *t* test. Bars represent the means ± SE.

Injection of *dsTreh1* into 3L3d larvae reduced *Treh1* transcripts by 78.12% (Figure 4A), demonstrating high interference efficiency. This silencing led to a 55.5% increase in trehalose content (Figure 4B), similar to the metabolic shift observed in *miR*-*2788* agomir-treated larvae. Furthermore, the expression of downstream chitin biosynthetic genes, including *CHI*, *CHS*, and *GPI*, was significantly downregulated, while *HK* expression was upregulated, potentially as a compensatory response to the elevated trehalose levels (Figure 4C).

The physiological impact of *Treh1* silencing was notable. The pupation rate decreased to 39%, representing a 56% reduction compared to the control group (88.7%). Similarly, the adult emergence rate declined to 18.3% compared to 81% in the control—a total reduction of 80.3% (Figure 4D). These results support that the phenotypes induced by *miR*-*2788* are at least partially mediated through its target, *Treh1*. Phenotypic observations revealed that *Treh1*-silenced larvae were unable to complete pupation properly; the resulting pupae were significantly smaller in size and displayed stunted growth compared to the *dsGFP* group (Figure 4E). The body length of *Treh1*-silenced larvae was significantly reduced compared to the control group (Figure 4F). The high degree of consistency between the *Treh1* RNAi phenotypes and the *miR*-*2788* agomir-induced defects provides further evidence that the *miR*-*2788*/*Treh1* axis is involved in the metamorphosis of *G. daurica*.

### 3.5. 20-Hydroxyecdysone (20E) Orchestrates the miR-2788/Treh1 Axis to Promote Metamorphosis

**Figure 5:**
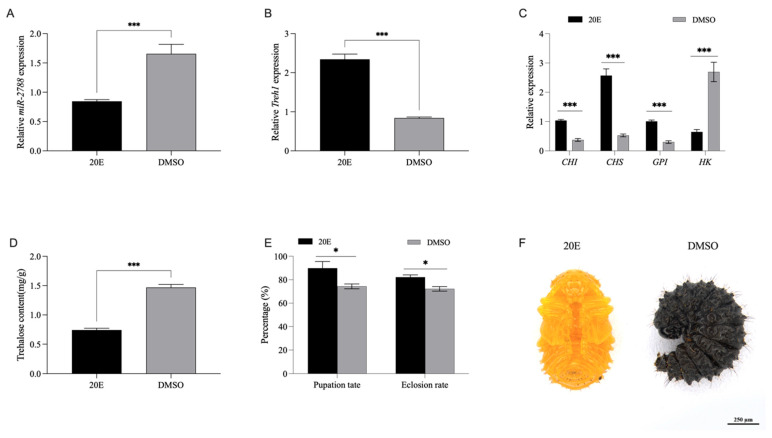
20E orchestrates the *miR*-*2788*/*Treh1* axis to promote metamorphosis. (**A**,**B**) Hormonal regulation of *miR*-*2788* (suppression) 20E (M = 0.85, SD = 0.08); DMSO (M = 1.66, SD = 0.46), t(7.41) = −4.95, *p* < 0.001 and *Treh1* (activation) 20E (M = 2.34, SD = 0.38); DMSO (M = 0.84, SD = 0.07), t(7.42) = 11.05, *p* < 0.001 following 20E treatment. (**C**) Stimulatory effects of 20E on the expression of downstream chitin biosynthetic pathway genes (*CHI*, *CHS*, *GPI*, and *HK*). (**D**) Reduction in endogenous trehalose content following 20E treatment. (**E**) Pupation and adult emergence rates in 20E-treated larvae compared to the control. (**F**) Representative images showing 20E-induced precocious pupation. Relative expression levels of *miR*-*2788* and *Treh1* were normalized to *U6* and *SDHA*, respectively, using quantitative real-time polymerase chain reaction (qRT-PCR). * and *** represent the significant difference at *p* < 0.05 and 0.001, by Student’s *t* test. Bars represent the means ± SE.

Insect molting and metamorphosis are regulated by the coordination of various hormones, with 20-hydroxyecdysone (20E) serving as a key ecdysteroid regulating larval growth and pupation. To determine whether the *miR*-*2788*/*Treh1* axis is subject to 20E regulation, we performed hormonal induction assays in 3L3d larvae of *G. daurica*. At 48 h post injection of 20E, *miR*-*2788* was downregulated by 48.9%, while *Treh1* was upregulated 1.78-fold (Figure 5A,B). These results suggest that 20E may negatively regulate *miR*-*2788* to derepress the expression of *Treh1*.

Since chitin biosynthesis is important to the molting process, we analyzed the expression of downstream chitin biosynthetic enzymes following 20E treatment. 20E injection significantly upregulated *CHI*, *CHS*, and *GPI*, while *HK* was downregulated (Figure 5C), indicating a shift from basal glycolysis toward chitin production.

Consistent with its role in regulating developmental timing and modulating metabolism, exogenous 20E treatment led to a 49.4% reduction in endogenous trehalose levels (Figure 5D), indicating enhanced trehalose mobilization for chitin synthesis. 20E-treated larvae entered the wandering stage earlier (Figure 5E), with the pupation rate increasing to 89.9% (vs. 74.4% in control) and the emergence rate rising to 82.2% (vs. 72.2% in control) (Figure 5E). These findings delineate a signaling cascade where 20E coordinates carbohydrate metabolism and chitin biosynthesis through the *miR*-*2788*/*Treh1* axis to facilitate successful metamorphosis (Figure 5F).

### 3.6. Validamycin Inhibition of Treh1 Triggers a Compensatory Negative Feedback Response of miR-2788

**Figure 6:**
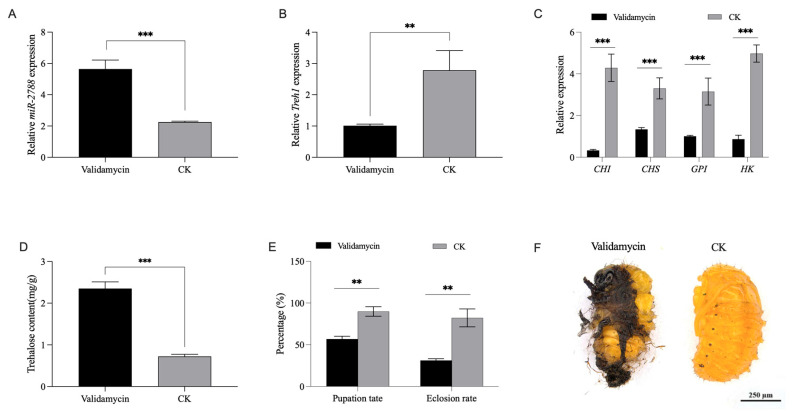
Validamycin inhibition of *Treh1* activity triggers a compensatory response of *miR*-*2788*. (**A**,**B**) Regulatory effects of Validamycin on the expression of *miR*-*2788* (Validamycin: (M = 5.86, SD = 1.92); CK: (M = 2.26, SD = 0.19), t(10.18) = 6.19, *p* < 0.001) and *Treh1* (Validamycin: (M = 1.02, SD = 0.18); CK: (M = 2.36, SD = 1.72), t(11.26) = −2.68, *p* = 0.0015). (**C**) Inhibitory effects of Validamycin on the expression of key genes in the chitin biosynthetic pathway (*CHI*, *CHS*, *GPI*, and *HK*). (**D**) Accumulation of endogenous trehalose following Validamycin treatment. (**E**) Pupation and adult emergence rates in Validamycin-treated larvae compared to the control. (**F**) Representative images of molting deformities and pupal defects induced by Validamycin treatment. Relative expression levels of *miR*-*2788* and *Treh1* were normalized to *U6* and *SDHA*, respectively, using quantitative real-time polymerase chain reaction (qRT-PCR). ** and *** represent the significant difference at *p* < 0.01 and 0.001, by Student’s *t* test. Bars represent the means ± SE.

Validamycin is a potent competitive inhibitor of trehalase and a critical tool for studying carbohydrate metabolism in insects. To investigate the pharmacological impact of *Treh1* inhibition on the *miR*-2788/*Treh1* axis, we performed microinjection experiments in 3L3d larvae of *G. daurica*. At 48 h post injection, Validamycin treatment led to a 41.3% downregulation of *Treh1* (Figure 6B) and unexpectedly triggered a 1.5-fold increase in *miR*-*2788* expression (Figure 6A). This suggests a possible compensatory response, where the reduction in trehalase activity is associated with the upregulation of *miR*-*2788*. As a suspected indirect inhibitor of chitin synthesis, Validamycin significantly disrupted the chitin biosynthetic pathway. Quantitative analysis showed that *CHI*, *CHS*, *GPI*, and *HK* were all significantly downregulated (Figure 6C). This broad suppression likely reflects cellular resource conservation or a consequence of glucose deprivation due to blocked trehalose hydrolysis.

The biochemical and physiological consequences of Validamycin treatment impacted larval development. The internal trehalose content increased 2.25-fold compared to the control group (Figure 6D), suggesting a potential disruption in carbohydrate utilization. Phenotypic observations revealed that Validamycin-treated larvae were unable to complete molting, frequently resulting in severe pupal deformities and developmental arrest (Figure 6F). Consequently, the pupation rate significantly decreased to 56.7% (vs. 89.9% in control), and the emergence rate dropped to 31.1% (vs. 82.2% in control) (Figure 6E). These pharmacological results are similar to the effects of *miR*-*2788* overexpression and *Treh1* RNAi, further supporting the involvement of the *miR*-*2788*/*Treh1* axis in the regulation of beetle metamorphosis.

## 4. Discussion

The successful metamorphosis of insects depends on a dynamic equilibrium between chitin biosynthesis and degradation, a process regulated by complex interplays between hormonal signaling and metabolic pathways. In this study, we characterized a potential molecular axis in *G*. *daurica* during the critical larval-to-pupal transition: a 20E-mediated *miR-2788/Treh1* pathway. This axis appears to function as a regulatory valve, contributing to the allocation efficiency of trehalose metabolism toward the chitin biosynthetic route. Given the technical challenges of rescue experiments in univoltine *G. daurica*, the phenotypic consistency observed across *miR*-*2788* overexpression, Treh1 RNAi, and pharmacological inhibition suggests a functional role of this regulatory axis.

For decades, 20E has been recognized as a key hormonal signal for molting and pupation, driving major developmental transitions through its nuclear receptor complex [31,32]. However, transcriptional regulation alone may not fully explain the rapid fluctuations and spatial precision of protein expression during development. Recently, microRNAs (miRNAs) have emerged as important post-transcriptional buffers that may mediate hormonal signaling [21]. Our observation that exogenous 20E treatment leads to the downregulation of *miR*-*2788* expression (Figure 5A) aligns with findings in other insect orders, where 20E negatively regulates specific miRNAs to modulate key metabolic genes. For instance, in *Nilaparvata lugens*, 20E-responsive *miR-8-5p* and *miR-2a-3p* were shown to target trehalase and phosphoacetylglucosamine mutase to modulate chitin biosynthesis [33]. This conserved Hormone-miRNA-Target gene cascade likely provides a mechanism to ensure the rapid upregulation of metabolic enzymes to meet the energy demands of pupation.

As the initial rate-limiting enzyme in chitin biosynthesis, the functionality of *Treh1* is important to the partitioning of carbohydrate resources. In insects, trehalose is not only the primary blood sugar providing an instant energy source but also the fundamental precursor for chitin synthesis [14,34]. Through dual-luciferase assays and in vivo functional validations, we confirmed that *miR*-*2788* directly targets and suppresses *Treh1* (Figure 1 and Figure 2). When *miR*-*2788* was overexpressed or *Treh1* was silenced via RNAi, larvae exhibited marked trehalose accumulation alongside the downregulation of core chitin biosynthetic genes such as *CHI* and *CHS* (Figure 3 and Figure 4). These results are consistent with the theory that trehalose hydrolysis is a contributing factor for chitin synthesis [35,36]. Notably, the compensatory upregulation of hexokinase (*HK*) in *Treh1*-deficient individuals (Figure 3A and Figure 4C) may reflect a metabolic adaptation. By enhancing glucose phosphorylation efficiency, the insect potentially attempts to maintain energy homeostasis, though this compensation is ultimately insufficient to prevent molting failure. Interestingly, the small-size phenotype was exclusively observed in *Treh1* RNAi larvae, whereas *miR*-*2788*-overexpressing individuals exhibited metamorphic arrest with unaltered body dimensions. Such discrepant outcomes potentially stem from the varying degrees of transcript silencing. The robust depletion of Treh1 by RNAi likely triggers a systemic energy deficit, thereby restricting larval biomass accumulation. Conversely, *miR*-*2788* acts as a rheostat-like regulator, providing nuanced modulation that is sufficient to perturb the sensitive metamorphic window but insufficient to arrest general ontogeny.

It should be noted that while a rescue experiment is often considered a definitive approach for miRNA functional validation, such an approach is technically prohibitive in *G. daurica* due to its univoltine lifecycle and high sensitivity to repeated microinjections. However, the consistent phenotypes observed across *miR*-*2788* overexpression, *Treh1* RNAi, and Validamycin treatment provide support for the *miR*-*2788*/*Treh1* axis, demonstrating that independent disruptions of this metabolic node lead to equivalent developmental outcomes.

Furthermore, our experiments using Validamycin suggest a potential regulatory feedback response. Validamycin not only recapitulated the malformations induced by *miR*-*2788* overexpression but was also associated with an increase in *miR*-*2788* levels (Figure 6A). This phenomenon suggests the possibility of a feedback mechanism in *G. daurica*. When *Treh1* activity is blocked, the resulting abnormal surge in intracellular trehalose may act as a metabolic signal, potentially through nutrient-sensing pathways (e.g., TOR or AMPK) to modulate miRNA processing [37,38]. This negative feedback response might serve to prevent osmotic damage caused by excessive trehalose fluctuations [39], revealing that insect developmental programs are governed not only by preset hormonal pulses but also by dynamic modifications based on endogenous metabolite levels [40]. Our findings demonstrate that Validamycin treatment modulates the expression levels of both *Treh1* and *miR*-*2788*. The metabolic signals generated following *Treh1* inhibition—including the systemic accumulation of trehalose and the significant downregulation of chitin biosynthetic genes (*CHI*, *CHS*, and *GPI*)—may act as a retrograde signal that influences the activity of downstream transcription factors. Similar systemic effects have been documented in *Nilaparvata lugens* and *Spodoptera frugiperda*, where Validamycin-induced trehalase inhibition triggered a broad reshuffling of gene expression across reproductive and metabolic pathways [41,42]. This feedback regulation likely integrates multiple regulatory layers, including nutrient-sensing pathways (e.g., AMPK or TOR), hormonal fluctuations, and potentially epigenetic modifications, to coordinate the insect’s physiological response to metabolic stress.

From the perspective of pest management, the discovery of the *miR*-*2788*/*Treh1* axis offers potential biotechnological insights. As a major pest of desert grasslands, *G. daurica* possesses developmental vulnerabilities at every metamorphic transition. The significant mortality and molting defects observed in our study (Figure 3, Figure 4 and Figure 6) underscore the importance of chitin homeostasis. As reviewed by Arakane and Muthukrishnan (2010), disrupting the delicate balance of chitin metabolism—whether through degradation or biosynthesis—is considered a potential strategy for developing intervention methods [43]. While the development of RNAi-based biopesticides faces practical challenges, the species-specificity of miRNA sequences suggests a direction for more precise management. Future research into delivery technologies, such as nanoparticle-mediated administration, may eventually help address current efficacy and stability issues [44]. By leveraging these molecular insights, it may be possible to achieve potent suppression of *G. daurica* while minimizing risks to non-target organisms, thereby supporting the development of integrated pest management programs in fragile desert grassland ecosystems.

In summary, this study proposes a molecular model: rising 20E levels are associated with suppressed *miR*-*2788* transcription, thereby promoting *Treh1* expression to drive the conversion of trehalose for chitin synthesis. When this balance is disrupted, the resulting metabolic changes lead to developmental arrest. These findings contribute to our understanding of how hormones and non-coding RNAs collaborate to regulate carbohydrate metabolism and identify potential targets for the development of management strategies against *G. daurica*.

## 5. Conclusions

In this study, we identified a 20-hydroxyecdysone-responsive *miR*-*2788*/*Treh1* regulatory axis in *Galeruca daurica*. We observed that miR-2788 targets *Treh1*, an enzyme involved in trehalose hydrolysis. 20E negatively regulates *miR*-*2788*, thereby modulating *Treh1* to support trehalose mobilization and chitin biosynthesis during metamorphosis. Disruption of this axis leads to trehalose accumulation, chitin deficiency, and developmental defects. Collectively, our findings suggest a hormonal–miRNA–metabolic cascade that contributes to the allocation of carbohydrate resources for chitin synthesis. The *miR-2788/Treh1* axis represents a potential molecular target for developing eco-friendly intervention strategies against this grassland pest.

## Data Availability

The original contributions presented in this study are included in the article/Supplementary Materials. Further inquiries can be directed towards the corresponding authors.

## Supplementary Materials

The following supporting information can be downloaded at https://www.mdpi.com/article/10.3390/insects17050502/s1, Table S1: primer sequence.
